# Reporting Incidents in the Psychiatric Intensive Care Unit: A Retrospective Study in an Italian University Hospital

**DOI:** 10.1097/NMD.0000000000001504

**Published:** 2022-04-08

**Authors:** Federica Mele, Luigi Buongiorno, Domenico Montalbò, Davide Ferorelli, Biagio Solarino, Fiorenza Zotti, Felice Francesco Carabellese, Roberto Catanesi, Alessandro Bertolino, Alessandro Dell'Erba, Gabriele Mandarelli

**Affiliations:** ∗ Section of Legal Medicine, Interdisciplinary Department of Medicine; † Department of Basic Medical Science, Neuroscience and Sense Organs; ‡ Section of Criminology and Forensic Psychiatry, Interdisciplinary Department of Medicine, University of Bari Aldo Moro, Policlinico di Bari Hospital, Bari, Italy.

**Keywords:** Incident reporting system, psychiatric intensive care unit, aggression, neurodevelopmental disorder

## Abstract

To evaluate the characteristics of the reported workplace violence in a psychiatric intensive care unit (PICU) by analyzing an electronic hospital incident reporting system (IRS). One hundred thirty reports were retrieved from January 2017 to June 2020, referring to assaults committed by patients (71% males) with an average age of 29.8 years (SD, 14.9). The most frequent psychiatric diagnosis was a neurodevelopmental disorder (33%). Physical aggression (84%) was more frequent than the other types of aggression. Nurses and unlicensed assistive personnel were the most frequent victims (65%). Aggressions were more frequent on Friday (18%) and between 4 p.m. and 8 p.m. (35%). A total of 64.9% of the incidents happened in the first 5 days of hospitalization. A significant association between physical aggression and diagnosis of neurodevelopmental disorder emerged. IRS could be helpful to identify high-risk patient groups and develop clinical strategies to reduce adverse events in clinical practice.

Clinical risk management plays a relevant role in health care systems due to the possible impact on the optimization of clinical results and the reduction of poor outcomes (Rodriguez et al., 2017). Hospital incident reporting system (IRS) is a method to systematically collect reports of adverse events by health personnel, including near miss and sentinel events (Elkin et al., 2016; Institute of Medicine, 1999; Kroll et al., 2018; Pham et al., 2013; Ramírez et al., 2018). Because IRS proved to help identify and reduce risks in health organizations, the implementation of hospital IRS has been encouraged by major health care leadership organizations such as the Joint Commission (Giesbrecht and Au, 2016), the Institute of Medicine (Institute of Medicine, 1999), and the World Health Organization (Larizgoitia et al., 2013).

Despite such premises, the underreport of adverse events through the IRS is an acknowledged problem, especially among physicians (Elkin et al., 2016; Farley et al., 2008; Hamilton et al., 2018), with estimates of less than 10% of adverse events being reported through the IRS (Classen et al., 2011; Kroll et al., 2018). Among the reasons leading clinicians to underreport adverse events are fear of possible negative consequences, lack of time, and insufficient knowledge of the IRS (Appelbaum et al., 2016; Mitchell et al., 2016). The use of information technology systems aimed at collecting events in the hospital was associated with an improvement in reporting rates (Elliott et al., 2014).

Aggressive behavior among patients hospitalized for psychiatric disorders is one of the leading causes of adverse events in nonsurgical environments (Mills et al., 2018). Psychiatric wards are at the highest risk to report adverse events (Catanesi et al., 2010; Palumbo et al., 2016), especially workplace violence (WPV) and suicide (Aguglia et al., 2020).

The use of the IRS would improve the capacity to collect information on incidents in this field. WPV is defined as an incident where a professional faces any sort of violence such as physical assault, verbal harassment, or any other type of aggression (Bizzarri et al., 2020; Ferri et al., 2016). A high rate of aggression toward medical doctors has been reported in some reviews with rates up to 70% (Elston and Gabe, 2016; Hills et al., 2012; Raveel and Schoenmakers, 2019; Vorderwülbecke et al., 2015). Although the prevalence of WPV is frequent in medical settings, some geographical differences are reported in the literature. Prevalence ranges from 54% in Thailand to 70% in Morocco. Some studies have shown an increasing trend in Asian countries and a decreasing trend in North America (Kumari et al., 2020; Liu et al., 2019). Furthermore, WPV can cause a reduction in the quality of patient care, relevant psychological consequences on the victims, and a decrease in job satisfaction (Bizzarri et al., 2020; Catanesi et al., 2016).

In Italy, the Ministerial recommendations to prevent violence (Ministero della Salute, 2007) and Law 113 of 2020 were issued to reduce the rates of aggression toward operators in the health care setting. From this perspective, many hospitals have implemented their risk assessment system with procedures including IRS to report any kind of sentinel event, including WPV (Ferri et al., 2016).

Although violence in psychiatric wards is frequently reported in the literature from an epidemiological and clinical point of view, this issue is scarcely investigated from a clinical risk management perspective. This study aims to analyze an electronic hospital IRS to evaluate the characteristics of WPV in a psychiatric intensive care unit (PICU) and the performance of the IRS system through the identification of any possible factors associated with aggressions, including psychiatric diagnosis, psychiatric symptoms severity, voluntary/involuntary hospitalization, and victim type.

These data could be helpful for the implementation of the IRS system and for clinical practice to develop corrective actions to prevent aggression in at-risk hospital wards.

## METHODS

### Eligibility and Inclusion Criteria

Clinical Risk and Patient Safety Management Unit of the Policlinico of Bari Hospital adopted IRS in 2015, and this system has been definitively implemented since January 2017. This system receives data from all the hospital wards (>1500 beds; >50,000 hospitalizations/year) (Ferorelli et al., 2020). Since the focus of the present study was aggressive behavior in the psychiatric ward, inclusion criteria were 1) reports of incidents that occurred in the adult PICU from January 1, 2017, to June 30, 2020, and 2) incident reports referring to patients' aggressive behavior.

The hospital IRS forms did not include data about psychiatric symptoms severity, including Brief Psychiatric Rating Scale (BPRS) (Ventura et al., 1993) total score at admission, length of hospitalization, age, education, psychiatric diagnosis retrieved from the discharge diagnosis, voluntariness of hospitalization, lifetime history of alcohol or drug abuse, previous psychiatric admission, and number and average hour of physical restrain. To retrieve such data, we subsequently performed further data collection from the patients' medical records related to each IRS form. Psychiatric diagnosis was based on *DSM-5* classification criteria (American Psychiatric Association, 2013).

To provide a qualitative measure of the patients’ aggressive behavior, we used the Modified Overt Aggression Scale (MOAS) (Margari et al., 2005) subscales, that is, verbal aggression, aggression against property, autoaggression, and physical aggression (Knoedler, 1989). The MOAS subscales were compiled only for evaluating the presence/absence of the specific behavior because the IRS did not provide sufficient information to retrospectively calculate the MOAS subscale scores. In the statistical analysis, the different types of aggression, even described in the same incident record, were considered as independent variables. Other data extracted from the IRS were the health care worker reporting the event, sex, and type of victim (physician, nurse, or other health care professionals, security guard, other patients, other people); number of victims; patient involuntary hospitalization; severity of the consequences (grading the severity from 0, no consequences, to 4, death); containment methods (physical, pharmacological); and number and average hour of physical restraint.

Data collection and analysis were carried out by two health workers from the clinical risk management unit and a psychiatrist from PICU. In the study period, 463 IRS forms were reported to the clinical risk management unit. On the total of the IRS reports, 139 were from the PICU.

### Data Collection and Analysis

Statistical analyses were performed using the Statistical Software for Social Sciences v. 20.0 (IBM SPSS Statistics for Windows, Version 20.0; IBM Corp, Armonk, NY). The alpha value was set to 0.05; all tests were two-tailed. Differences between groups in continuous variables were analyzed by the independent sample *t*-test. The chi-square test or Fisher’s exact test was used for comparisons between categorical variables.

## RESULTS

The initial search yielded a total of 139 IRS forms in the study period, which were further screened for the presence of aggressive behavior, from which 130 reports fully satisfied our inclusion criteria. Of the *n* = 130 reports analyzed, 16.9% were related to incidents that happened in 2017, 54.6% in 2018, 17.7% in 2019, and 10.8% in the first semester of 2020. The average age of patients who determined the incident reports was 29.8 years (SD, 14.9), and most of them were male 71.5%. The most common psychiatric diagnoses were neurodevelopmental disorders (33%), schizophrenia spectrum disorders (24%), and bipolar disorders (23%). In our sample, 43% of patients had a history of substance use or addiction. The BPRS total score at the admission was, on average, 58.3 (SD, 11.9), and 20.7% of the patients were involuntarily hospitalized when the incident occurred (see Table 1). Independent sample *t*-test disclosed a higher BPRS total score at admission in those patients who would have generated a physical assault report during hospitalization, compared with those who did not (59.3 ± 11.5 *vs*. 52.4 ± 13.3, *p* < 0.05). Patients who generated a report for verbal aggression showed lower BPRS total scores at admission (55.2 ± 12.9 *vs*. 59.9 ± 11.2, *p* < 0.05); no differences were found for autoaggression and property aggression.

**TABLE 1:** Clinical Characteristics of the Patients Who Generated Incident Reports From the Psychiatric Intensive Care Unit Between 2017 and June 2020

		Min	Max	Mean	Standard Deviation
Length of hospitalization (in days)		5	139	55.1	52.4
BPRS		28	98	58.3	11.9
Diagnosis				Principal, *n* (%)	Secondary, *n* (%)
Schizophrenia spectrum disorders				31 (26.9)	0
Bipolar disorders				30 (26.0)	0
Personality disorders				1 (0.9)	11 (9.6)
Substance-related and addictive disorders				5 (4.4)	4 (3.5)
Neurodevelopmental disorders				43 (37.4)	14 (12.2)
Neurocognitive disorders				1 (0.9)	2 (1.7)
Disruptive, impulse control and conduct disorders				4 (3.5)	38 (33.0)
Obsessive-compulsive disorder				0	1 (0.9)
No secondary diagnosis				0	45 (39.1)
History of substance use				*n* (%)	
Cannabis				25 (19.2)	
Psychostimulants				13 (10)	
Alcohol				18 (13.8)	
No history of substance use				77 (59.2)	
NA				20 (15.4)	
Previous psychiatric admission				*n* (%)	
Yes				89 (68.5)	
No				41 (31.5)	
Involuntary hospitalization				*n* (%)	
Yes				23 (20.7)	
No				88 (79.3)	
Aggression during involuntary hospitalization				*n* (%)	
Yes				20 (18)	
No				91 (82)	
Physical restraint				*n* (%)	
Yes				69 (62.2)	
No				42 (37.8)	
Physical restraint after aggression				*n* (%)	
Yes				41 (36.9)	
No				70 (63.1)	
		Min	Max	Mean	Standard Deviation
Average of physical restraint (in min)		47	1202	390.7	246.3

Note: Data were missing for length of hospitalization (*n* = 18). Data were missing for BPRS (*n* = 20). Data were missing for involuntary hospitalization, aggression during involuntary hospitalization, physical restraint, physical restraint after aggression (*n* = 19). Data were missing for psychiatric diagnosis (*n* = 15).

NA, not assessed, missing data.

Aggressions were more frequent on Friday (17.7%) and in the time slot between 4 p.m. and 8 p.m. (34.6%) (Table 2). We found that 64.9% of the incidents happened in the first 5 days of hospitalization or after 20 days from admission (Table 2). The average duration of hospitalization at the time of the incident was 23.7 days (SD, 11.9).

**TABLE 2:** Day and Time Characteristics of the Incident Reports From the Psychiatric Intensive Care Unit Between 2017 and June 2020

	*n* (%)
Day of the week	
Sunday	20 (15.4)
Monday	16 (12.3)
Tuesday	14 (10.8)
Wednesday	17 (13.1)
Thursday	22 (16.9)
Friday	23 (17.7)
Saturday	18 (13.8)
Time interval	
0:00–3:59 a.m.	7 (5.5)
4:00–7:59 a.m.	5 (3.8)
8:00–11.59 a.m.	26 (20)
12:00 a.m.–3:59 p.m.	32 (24.6)
4:00–7:59 p.m.	45 (34.6)
8.00–11.59 p.m.	15 (11.5)
Days from the admission	
≤5	33 (29.8)
6–10	22 (19.8)
11–15	10 (9)
16–20	7 (6.3)
≥20	39 (35.1)
Reporter	
Nurse	34 (26.2)
Doctor	96 (73.8)
Health care worker	0 (0)

Note: Data were missing for days from the admission (*n* = 19).

The victims’ characteristics are described in Table 3. Physical aggressions were more frequent than the other types of aggression (83.8%) and frequently (67.4%) co-occurred with verbal aggression or immediately thereafter. Health care workers, including nurses and unlicensed assistive personnel, were the most frequent victims (65.4%).

**TABLE 3:** Type of Aggression and Victim Characteristics of the Incident Reports From the Psychiatric Intensive Care Unit Between 2017 and June 2020

		Min	Max	Mean	Standard Deviation
Victim number		0	3	1.24	0.745
Type of aggression, *n* (%)					
Verbal aggression			46 (35.4)		
Properties aggression			16 (12.3)		
Autoaggression			7 (5.4)		
Physical aggression			109 (83.8)		
Victim type, *n* (%)					
Patient			27 (20.8)		
Doctor			32 (24.6)		
Health care worker			85 (65.4)		
Guard			14 (10.8)		
Visitor			3 (2.3)		
Severity of injury, *n* (%)					
No injury			36 (27.7)		
Minor			56 (43.1)		
Medium			35 (26.9)		
Serious			3 (2.3)		
Death			0		

Chi-square analysis disclosed that, among those who committed property aggression, there was a higher percentage in the male group of patients than in the female group of patients (15.1% *vs*. 5.9%, *p* < 0.05) (Table 4). The female group of patients showed a higher percentage of physical aggression than the male group (91.2% *vs*. 81.7%, *p* < 0.05) (Table 4). No other significant differences were detected considering the aggressor's sex.

**TABLE 4:** Sex Differences in Different Type of Aggression and Victim

	Total (*n* = 127)	Males (*n* = 93)	Females (*n* = 34)	*p*
Type of aggression				
Verbal aggression, *n* (%)				NS^a^
Yes	45 (35.4)	33 (35.5)	12 (35.3)	
No	82 (64.6)	60 (64.5)	22 (64.7)	
Properties aggression, *n* (%)				<0.05^a^
Yes	16 (12.6)	14 (15.1)	2 (5.9)	
No	111 (87.4)	79 (84.9)	32 (94.1)	
Autoaggression, *n* (%)				NS^a^
Yes	5 (3.9)	4 (4.3)	1 (2.9)	
No	112 (96.1)	89 (95.7)	33 (97.1)	
Physical aggression, *n* (%)				<0.05^a^
Yes	107 (84.3)	76 (81.7)	31 (91.2)	
No	20 (15.7)	17 (18.3)	3 (8.8)	
Victim type				
Patient, *n* (%)				NS^a^
Yes	27 (21.3)	23 (24.7)	4 (11.8)	
No	100 (78.7)	70 (75.3)	30 (88.2)	
Doctor, *n* (%)				NS^a^
Yes	32 (25.2)	26 (28)	6 (17.6)	
No	95 (74.8)	67 (72)	28 (82.4)	
Health care worker, *n* (%)				NS^a^
Yes	83 (65.4)	58 (62.4)	25 (73.5)	
No	44 (34.6)	35 (37.6)	9 (26.5)	
Guard, *n* (%)				NS^a^
Yes	14 (11)	13 (14)	1 (2.9)	
No	113 (89)	80 (86)	33 (97.1)	
Visitor, *n* (%)				NS^a^
Yes	3 (2.4)	1 (1.1)	2 (5.9)	
No	124 (97.6)	92 (98.9)	32 (94.1)	

^a^*p* value by chi-square; *n*, 3 data were missing for patients' sex.

NS indicates not significant.

The analysis of the possible association between type of aggression and psychiatric diagnosis showed a significant association between physical aggression and a discharge diagnosis of neurodevelopmental disorder (Table 5). However, all these patients were admitted because of concomitant agitation.

**TABLE 5:** Diagnosis Differences in Different Type of Aggression and Victim

	Total	Schiz	Bip	Depr	Pers	Add	Neu_d	Neu_c	Dis	*p*
Type of aggression	n = 115	n = 28	n = 31	n = 1	n = 1	n = 3	n = 46	n = 1	n = 4	
Verbal aggression, *n* (%)										NS^a^
Yes	40 (34.8)	14 (50)	11 (35.5)	1 (100)	0	1 (33.3)	11 (23.9)	0	2 (50)	
No	75 (65.2)	14 (50)	20 (64.5)	0 (0)	1 (100)	2 (66.7)	35 (76.1)	1 (100)	2 (50)	
Properties aggression, *n* (%)										NS^a^
Yes	13 (11.3)	1 (3.6)	5 (16.1)	0	1 (100)	0	5 (10.9)	0	1 (25)	
No	102 (88.7)	27 (96.4)	26 (83.9)	1 (100)	0	3 (100)	41 (89.1)	1 (100)	3 (75)	
Autoaggression, *n* (%)										NS^a^
Yes	3 (2.6)	1 (3.6)	1 (3.2)	0	0	0	1 (2.2)	0	0	
No	112 (97.4)	27 (96.4)	30 (96.8)	1 (100)	1 (100)	3 (100)	45 (97.8)	1 (100)	4 (100)	
Physical aggression, *n* (%)										<0.05^a^
Yes	97 (84.3)	21 (75)	25 (80.6)	1 (100)	0	3 (100)	44 (95.7)	1 (100)	2 (50)	
No	18 (15.7)	7 (25)	6 (19.4)	0	1 (100)	0	2 (4.3)	0	2 (50)	
Victim type	n = 115	n = 28	n = 31	n = 1	n = 1	n = 3	n = 46	n = 1	n = 4	
Patient, *n* (%)										NS^a^
Yes	23 (20)	6 (21.4)	7 (22.6)	0	0	1 (33.3)	9 (19.6)	0	0	
No	92 (80)	22 (78.6)	24 (77.4)	1 (100)	1 (100)	2 (66.7)	37 (80.4)	1 (100)	4 (100)	
Doctor, *n* (%)										NS^a^
Yes	31 (27)	6 (21.4)	5 (16.1)	1 (100)	0	0	19 (41.3)	0	0	
No	84 (73)	22 (78.6)	26 (83.9)	0	1 (100)	3 (100)	27 (58.7)	1 (100)	4 (100)	
Health care worker, *n* (%)										NS^a^
Yes	78 (67.8)	20 (71.4)	17 (54.8)	1 (100)	0	2 (66.7)	35 (76.1)	1 (100)	2 (50)	
No	37 (32.2)	8 (28.6)	14 (45.2)	0	1 (100)	1 (33.3)	11 (23.9)	0	2 (50)	
Guard, *n* (%)										NS^a^
Yes	10 (8.7)	2 (7.1)	3 (9.7)	0	0	0	5 (10.9)	0	0	
No	105 (91.3)	26 (92.9)	28 (90.3)	1 (100)	1 (100)	3 (100)	41 (89.1)	1 (100)	4 (100)	
Visitor, *n* (%)										<0.05^a^
Yes	3 (2.6)	0	1 (3.2)	1 (100)	0	0	1 (2.2)	0	0	
No	112 (97.4)	28 (100)	30 (96.8)	0	1 (100)	3 (100)	45 (97.8)	1 (100)	4 (100)	

Note: Data were missing for psychiatric diagnosis (*n* = 15).

^a^*p* value by chi-square.

NS, not significant; Schiz, schizophrenia; Bip, bipolar; Depr, depression; Pers, personality disorders; Add, addiction; Neu_d, neurodevelopmental disorders; Neu_c, neurocognitive disorders; Dis, disruptive behavior.

## DISCUSSION

Workplace violence in psychiatric wards is widely debated in literature mainly from a clinical and epidemiological point of view but scarcely from that of risk management. The prevalence of WPV is an important problem in health care settings, and several corrective interventions would be needed to reduce its incidence. These could include more sense of responsibility of health care professionals in reporting adverse events through hospital reporting systems. Thanks to the analysis of IRS, this phenomenon could be evaluated also from a clinical risk management perspective (Civilotti et al., 2021; Kumari et al., 2020; Liu et al., 2019).

The analysis of IRS reported in PICU (*n* = 139) in the study period (from January 1, 2017, to June 30, 2020) allowed us to extract 130 reports related to aggressive behavior. Our IRS was not specifically designed to be implemented in psychiatric services. However, the PICU is the operational unit with the largest proportion of reports (Ferorelli et al., 2020). These events that occur most in the PICU such as assaults, falls, and suicides, are sentinel events that must be reported as indicated by the Italian ministerial recommendations (Ferracuti et al., 2021).

Most of our findings are consistent with existing data and provide also further insight into specific characteristics of sex and diagnosis association with aggressions in an acute psychiatric hospital setting. It is widely recognized that aggressions are perpetrated frequently by young males in psychiatric settings (Akopov et al., 1996; Amore et al., 2008; Ramesh et al., 2018), probably because of different interpersonal relationships involving the male population (Iozzino et al., 2015) as well as neurobiological factors (Repple et al., 2018). Moreover, men showed a positive association between orbitofrontal cortex, rectal gyrus, and anterior cingulate cortex activity in the provocation contrast and their tendency to respond aggressively (Repple et al., 2018).

Despite this existing evidence, our results showed a higher proportion of physical aggression in female patients than in males. The significant association between female sex and physical aggression in the incident reports of a single academic hospital PICU is an interesting result that deserves further analysis on larger samples. A possible interpretation of such a result might reside in health care personnel's greater attitude in reporting to a supervisory system an event that is perceived as infrequent or not corresponding to cultural stereotypes.

In our study, most of the aggressions occurred in the time interval between 4:00 p.m. and 8:00 p.m. and 60% between 12:00 a.m. and 8:00 p.m., in line with the results from the few similar studies that registered the time slot of the aggression in psychiatric settings (Bizzarri et al., 2020; Ferri et al., 2016). This result could be related to the lower number of at-work personnel during this time frame (Edward et al., 2014). We found no significant differences in the frequency of reports among the weekdays, although the evidence suggests a higher proportion of incidents during the weekend (Gacki-Smith et al., 2009).

Our results are in line with the existing evidence indicating a higher risk of aggressions during the first days of hospitalization as we found that almost 29.8% of the reports regarded the 5 days after admission (Barlow et al., 2000; Calegaro et al., 2014) and 35.1% after 20 days. A high percentage of incidents in the first 5 days might be explained by partial ineffectiveness of pharmacotherapy as well as that the acute psychiatric symptoms that lead to the hospitalization. The following increase of incidents after 20 days from admission might instead be associated with the development of maladjustment to a condition characterized by restrictions and limitations.

Psychiatric symptoms severity and specific psychiatric symptoms have been associated with the risk of patients' aggressive behavior (Amore et al., 2008; Calegaro et al., 2014). Our results are in accordance with those reported in the literature; for example, Amore et al. found that an average BPRS total score higher than 50 was significantly associated with violent behavior (Amore et al., 2008). The mean BPRS total score at the admission of the patients who generated incident reports included in our sample is 58.3 (SD, 11.9). This result confirms the need for particular attention and care dedicated to those patients with severe psychiatric symptoms in the light of clinical risk reduction.

History of addiction is an acknowledged risk factor for workplace aggression (Amore et al., 2008; Carabellese et al., 2013; Dack et al., 2013; Elbogen and Johnson, 2009; Ferri et al., 2016). Our results seem to confirm a general history of substance use in those patients who generated an incident report (43%). Nonetheless, in our study sample, a diagnosis of substance-related disorders was infrequent, a result that deserves further investigation to clarify the relationship among substance use history, diagnosis, and incident reports in the PICU.

In the present study, the rate of involuntary hospitalization among patients who generated an incident report was 20.7%, significantly higher than the estimated 10% of involuntary psychiatric hospitalization in the Italy PICUs (Ferracuti et al., 2021; Gaddini et al., 2005). There is evidence of the association between aggression and involuntary hospitalization (Abderhalden et al., 2007; Biancosino et al., 2009; Canova Mosele et al., 2018); nevertheless, dangerousness to self or others is not a legal criterion for involuntary psychiatric hospitalization in Italy. Our result of a large proportion of involuntarily hospitalized patients among those included is not unexpected, because a large study has already shown that approximately 10% of patients undergoing involuntary psychiatric hospitalization in Italy present reasons of aggression and danger (Ferracuti et al., 2021).

Neurodevelopmental disorders, schizophrenia spectrum disorders, and bipolar disorders were the most frequent diagnoses we found in our incident reports sample. The possible association between psychotic disorders and aggression to health professionals has already been highlighted, although several other factors might mediate such connection, including substance abuse (Blanco et al., 2018; Dack et al., 2013; Fazel et al., 2009; Iozzino et al., 2015). We found that the most frequent psychiatric diagnosis was a neurodevelopmental disorder. This result might be related to the reason for the admission, that is, concurrent agitation. Despite neurodevelopmental disorders having been associated with aggressive behavior (Rojahn et al., 2008; Schroeder and Courtemanche, 2012), they are rarely considered in connection with workplace aggression (Coid et al., 2016). We suggest that a major focus on this relevant issue, as well as on other neuropsychological factors, should be accounted for in further studies. Poor treatment response in neurodevelopmental disorders might also account for such results. The group of health care workers (nurses and unlicensed assistive personnel) was most frequently victimized, confirming a bulk of evidence (Edward et al., 2014, 2016; Martinez, 2016; Wei et al., 2016) also through the analysis of clinical risk management data.

Possible limitation of this study include the health care personnel tendency to underreport adverse events (Pham et al., 2013), a phenomenon that could have occurred and influenced the data analyzed in this study; however, our purpose was to analyze the IRS system reports. Although underreport is a common problem of IRS (Kroll et al., 2018), improving patient safety in different health settings also depends on the implementation of this tool.

The implementation of the IRS has been proposed as a valuable instrument to predict violence, especially in psychiatric wards (Ramesh et al., 2018). Since only the most serious events tend to be reported and no other sources of information were available, it was not possible to compare the events reported via the IRS with the actual number of assaults that occurred over the study period (Kroll et al., 2018). A multicenter study on 40 psychiatric units showed that almost 50% of the aggressions were also reported to the clinical risk management through an adverse event reporting system (Reilly et al., 2019). This evidence could be explained as psychiatrists tend to report violence informally to colleagues, considering WPV a normal consequence of psychiatric patient care (Kavanagh and Watters, 2010).

It is possible to hypothesize that the number of reports sent to the clinical risk management unit was lower than the actual number of incidents befell in the PICU, determining an underestimation of the incidents. It might also be due to the need for staff to fully develop a risk management perspective, in some cases modifying an established tendency to treat violence or risk events as naturally associated with clinical practice. Moreover, IRS could be perceived as punitive or as the bearer of professional liability issues. Better coordination between clinical risk management and medicolegal services has been proposed to improve IRS and reduce underreporting (Bolcato et al., 2019).

Nurses were the most frequent victims of aggression by patients (65.4%); nonetheless, they reported just 26.2% of the incidents we analyzed here compared with 73.8 of medical doctors. The discrepancy between victimization and the tendency to report incidents suggests the need for specific training in the use of the IRS in nurses.

The reporting of adverse events is particularly important in high-risk wards such as psychiatric departments where aggression to health care professionals is a matter of concern, also because more than 1/4 (29.2%) were medium or severe injuries, according to the IRS classification. Another limitation is the single-centered study design, with possible consequent selection bias due to social and/or psychopathological, limiting the generalizability of the results and deserves replication on larger and multicenter samples.

## CONCLUSIONS

The analysis of IRS data from an academic hospital PICU provided insight into the characteristics of the reported adverse events occurring in such a clinical setting. Physical and verbal assaults toward health care personnel by patients suffering from schizophrenia spectrum disorders or neurodevelopmental disorders proved to be the most frequently reported event. Reports of assaults by female patients in the PICU were higher than expected, indicating a possible specificity of such subgroup of acute psychiatric patients deserving attention by clinical risk managers.

The timing of the incidents, which were more frequent in the afternoon and the first 5 days after admission in the PICU, represents helpful information to develop specific management strategies to reduce the risks of adverse events. Possible clinical strategies to reduce adverse events in the PICU should also consider the foreseeable positive consequences of reducing involuntary psychiatric treatment and physical restraint.

It is possible to react proactively through pharmacological and psychological approaches, focusing on patients at higher risk for aggressive behavior. This proactive attitude could reduce events and lead to a reduction in involuntary hospitalizations and physical restrictions resulting from aggressive behaviors in hospital psychiatric care.

As concerning the IRS, this study highlighted the possible usefulness to implement the collection of psychiatric clinical data in the IRS, including diagnosis and coercive measures. Finally, the relatively low number of reported incidents suggests the need for specific training of the health care personnel to improve their attitude toward reporting adverse events.

## DISCLOSURE

The authors declare no conflict of interest.

This is an observational retrospective study. Therefore, our hospital ethics committee was not involved, and informed consent was not necessary. The study adheres to the Helsinki Declaration of Human Rights (1964), subsequently amended by the 64th WMA General Assembly (2013).

Federica Mele, ORCID ID: https://orcid.org/0000-0003-4289-8907

Luigi Buongiorno, ORCID ID: https://orcid.org/0000-0001-8757-088X

Domenico Montalbò, ORCID ID: https://orcid.org/0000-0003-0703-3318

Davide Ferorelli, ORCID ID: https://orcid.org/0000-0001-8150-7476

Biagio Solarino, ORCID ID: https://orcid.org/0000-0003-3096-8998

Felice Francesco Carabellese, ORCID ID: https://orcid.org/0000-0001-9942-6754

Roberto Catanesi, ORCID ID: https://orcid.org/0000-0003-1691-6366

Alessandro Bertolino, ORCID ID: https://orcid.org/0000-0002-1251-1380

Alessandro Dell'Erba, ORCID ID: https://orcid.org/0000-0003-3767-014X

Gabriele Mandarelli, ORCID ID: https://orcid.org/0000-0003-4887-5108
